# The Ultrahigh-Resolution Protein Crystal Structure of Crambin

**DOI:** 10.1063/4.0000934

**Published:** 2025-10-27

**Authors:** Changsoo Chang, Julian C. H. Chen, Miroslaw Gilski, Dominika Borek, Zbyszek Otwinowski, Maciej Kubicki, Mariusz Jaskolski, Andrzej Joachimiak

**Affiliations:** 1 Argonne National Laboratory, Lemont, IL, USA; 2 Los Alamos National Laboratory, Los Alamos, NM, USA; 3 Polish Academy of Sciences, Poznan, PL.WP, Poland; 4 Adam Mickiewicz University, Poznan, PL.WP, Poland; 5 The University of Texas Southwestern Medical Center, Dallas, TX, USA; 6 University of Chicago, Chicago, IL, USA

## Abstract

Ultra-high-resolution crystal structures of proteins provide critical insights into protein structure, dynamics, hydrogen bonding, and solvent networks. Crambin, a small hydrophobic storage protein consisting of 46 residues (4.7 kDa), is found in the embryonic tissue of seeds from *Crambe abyssinica*. This protein is renowned for its ability to crystallize readily, forming some of the best-ordered macromolecular crystals known, which diffract X-rays to the highest sub-atomic resolution recorded for any protein to date.

We have previously reported the room temperature structure of crambin, refined to an exceptional resolution of 0.70 Å using SHELXL. That analysis revealed intricate details of the dynamic solvent network, characterized by alternative side chain conformations and shifts in water molecule positions. In this work, we extend our investigation by presenting new structural data collected at cryogenic temperatures: 15K using liquid helium and 100K using liquid nitrogen cooling.

We will report the ultra-high-resolution structures at 15K and 100K, providing a comparative analysis of the solvent networks across these different temperature datasets. This comparison aims to deepen our understanding of the solvent and protein dynamics, offering valuable insights into the protein interactions within solvent environments. Our findings underscore the significance of ultrahigh-resolution crystallography in elucidating the complex interplay between proteins and their solvent environments, with potential implications for the broader field of structural biology.

